# Professional Re-education

**Published:** 1919-01

**Authors:** 


**Professional Re-education.** By Dr. Melis. *Revue Interalliée pour les Mutilés de la Guerre.* April, 1918.

Belgium has lost the greatest part of its workmen during this war. It will be a question of life or death for this State to make use of all its remaining energies. Therefore the professional re-education of " mutilés " is a problem of national importance for the Belgian government. Of course, the fact that nearly all of the country is still invaded renders more difficult a perfect organization.

The Belgian army possesses two orthopedic hospitals : one in *Bonsecours-les-Rouen*, the other in *Le Havre.* Professional re-education is practised in two schools : in *Port-Villez*, and *Le Havre.*

Dr. Melis insists on the fact that the mutilés are left completely free to choose whatever trade they prefer. Theoretically all disabled soldiers are obliged to undergo the re-educational training courses.

The author considers that professional re-education must be under the care of the Government, and must be continued after the war.

